# Chemical Composition and Bioactivity of *Nelumbo nucifera* Gaertn. Flower Extract Fractions: In Vitro Antioxidant and Anti-Inflammatory Properties

**DOI:** 10.3390/cimb47121065

**Published:** 2025-12-18

**Authors:** Jung Ha Choo, So Young Lee, Kyoungin Min, Nae Gyu Kang

**Affiliations:** Science Research Park, LG Household and Healthcare Ltd., Gangseo-gu, Seoul 07795, Republic of Korea; soyounglee@lghnh.com (S.Y.L.); kimin@lghnh.com (K.M.); ngkang@lghnh.com (N.G.K.)

**Keywords:** *Nelumbo nucifera*, lotus, flower, ethanol fraction, myricetin, quercetin derivatives, antioxidant, anti-inflammation

## Abstract

With the aging global population, interest in skin aging and skin health products is increasing. *Nelumbo nucifera* Gaertn. (lotus) has been widely used for its pharmacological benefits, including antioxidant, anti-inflammatory, skin-whitening, and anti-aging properties. In this study, we aimed to develop a safe and biologically active extract by extracting lotus flowers with hot water, followed by sequential fractionation using porous resin chromatography with stepwise ethanol elution (100% water and 30%, 70%, and 100% ethanol). The 30% and 70% ethanol fractions showed the highest total polyphenol and flavonoid contents. Liquid chromatography–electrospray ionization–mass spectrometry analysis identified major flavonoids, including myricetin and quercetin derivatives, in these fractions. These fractions were combined to formulate a novel *Nelumbo nucifera* flower extract (NFE), which exhibited potent antioxidant activity confirmed by 2,2-diphenyl-1-picrylhydrazyl, 2,2′-azinobis(3-ethylbenzothiazoline-6-sulfonic acid) and ferric reducing antioxidant power assays. NFE significantly inhibited nitric oxide and prostaglandin E_2_ secretion in lipopolysaccharide-activated murine RAW264.7 macrophages. In human keratinocytes HaCaT cells, NFE reduced tumor necrosis factor-α-induced expression and secretion of the pro-inflammatory cytokines interleukin (IL)-6 and IL-8 without cytotoxicity. These findings demonstrate that NFE has strong in vitro antioxidant and anti-inflammatory activities, supporting its potential as a bioactive ingredient for application in improving skin health preparations.

## 1. Introduction

An aging society is characterized by a population in which individuals aged 65 and older constitute a defined threshold proportion. This demographic transition results from declining birth and death rates combined with increased life expectancy. Currently, most people worldwide are expected to live beyond 60 years, leading to a steady rise in both the size and proportion of the older population [1]. As global aging accelerates, a growing interest in appearance and self-care exists, often driven by negative perceptions of aging and a strong desire to maintain youthfulness [2]. Unlike internal organ aging, which is not externally visible, skin aging provides a prominent and observable sign of chronological progression, motivating the use of cosmetics, dietary supplements, and pharmaceuticals to prevent or alleviate skin aging.

The skin, the body’s most extensive organ, acts as a crucial barrier against external environmental threats while playing an essential role in maintaining internal homeostasis and metabolic regulation. Skin aging arises from a combination of internal determinants—such as genetic factors, cellular and hormonal activity, and overall metabolic processes—and external contributors, including chronic ultraviolet (UV) radiation, environmental pollution, ionizing radiation, and exposure to chemicals and toxic substances [3].

Oxidative stress is a key driving force underlying skin aging caused by both internal and external factors. In chronological aging, mitochondrial oxidative metabolism—including ATP production from glucose—and mitochondrial dysfunction are major sources of reactive oxygen species (ROS). Extrinsic aging, on the other hand, involves excessive ROS generation resulting from redox imbalance induced by environmental stressors including UV light, air pollutants, cigarette smoke, and poor nutrition [4]. Oxidative stress damages fundamental cellular components in skin cells, including DNA, proteins, and lipids, thereby impairing their function. This damage accelerates the degradation of key structural proteins including collagen and elastin, manifesting as wrinkles, decreased elasticity, and skin laxity [5]. Inflammation is also a crucial contributor to skin aging, especially in photoaging induced by UV radiation, where inflammatory responses exacerbate the aging process. Chronic and repeated UV exposure causes cellular injury, fostering a persistent low-grade inflammatory state termed “inflammaging” [6]. This UV-driven inflammation activates signaling pathways including NF-κB and MAPK, which increase the secretion of inflammatory mediators and matrix metalloproteinases (MMPs), enzymes responsible for collagen and elastin degradation. Additionally, ROS generated following-UV exposure amplify inflammatory signaling, further accelerating skin aging [7].

Extracts from various plants exhibiting multiple pharmacological activities, notably antioxidant and anti-inflammatory effects, have been extensively incorporated into skincare products and treatments aimed at preventing or ameliorating skin aging [8]. Traditionally, natural plant-derived extracts have been used in dermatological care, with their efficacy and safety supported by long-standing empirical evidence. These extracts are obtained from diverse botanical parts—including whole plants, leaves, roots, flowers, and seeds—and contain an abundant concentration of bioactive compounds including polyphenols, carotenoids, tocopherols, peptides, and polysaccharides, all recognized for their potent antioxidant properties. These compounds provide multiple benefits, including free radical scavenging, anti-inflammatory effects, and promotion of skin regeneration, thereby effectively contributing to prevention of skin aging [9]. Owing to their established safety and functionality, natural plant-based materials are increasingly valued in the cosmetic, nutraceutical, and pharmaceutical industries.

*Nelumbo nucifera* Gaertn., commonly referred to as the lotus, is a water-dwelling plant species within the Nelumbonaceae family, widely cultivated throughout East Asia, including Korea, China, Japan, India, and Thailand [10]. This visually striking plant has been cultivated since antiquity, and its distinctive ability to bloom pristinely in muddy waters without staining has inspired its recognition as a sacred and spiritual symbol in various religious traditions [11]. Various parts of the lotus plant—including rhizomes, leaves, flowers, and seeds—are used in culinary applications and have been traditionally employed in folk medicine across multiple cultures to alleviate diverse medical issues such as sunstroke, diarrhea, dysentery, hemorrhoids, dizziness, bleeding disorders, skin conditions, infections, cough, hypertension, fever, urinary problems, and to promote conception [12,13].

*Nelumbo nucifera* has been widely used for its pharmacological benefits, including antioxidant, anti-inflammatory, skin-whitening, and anti-aging properties, such as attenuating wrinkles, preserving collagen, and limiting the formation of advanced glycation end-products [14,15,16,17,18,19]. Research in pharmacology has revealed that the lotus displays diverse bioactivities, such as antioxidant, antimicrobial, hepatoprotective, hypolipidemic, anti-obesity, hypoglycemic, and sleep-promoting effects [20,21,22,23,24,25,26,27,28]. These therapeutic properties of lotus are largely attributed to its natural bioactive compounds, particularly polyphenols such as flavonoids and phenolic acids, along with alkaloids. Chemical analyses of lotus extracts have revealed abundant flavonoid derivatives—including myricetin, kaempferol, and quercetin derivatives—alongside alkaloids such as neferine, liensinine, isoliensinine, lotusine, and pronudiferine [15,19,20,21,22,23,24,25,26,27,28,29,30,31,32].

Efficient extraction methods are critical to maximize the yield of pharmacologically active compounds from lotus. Approaches such as optimizing extraction solvents, techniques, and subsequent fractionation can significantly enhance the concentration of bioactive constituents in the extracts. Various organic solvents have been widely utilized to extract bioactive compounds from different parts of the lotus plant. However, some organic solvents possess toxic and irritant properties that may pose potential risks to human health. In particular, when applied to cosmetics and food products involving skin contact or ingestion, ensuring the complete removal of organic solvents after extraction is essential. Additionally, strict monitoring and control of residual solvent levels are required to guarantee safety. For these reasons, growing interest in safer alternatives such as water-based extraction methods is evident. However, water extraction generally results in lower concentrations of active compounds compared to organic solvents. Moreover, studies reporting improvement in the efficiency of water extraction combined with fractionation techniques to increase the yield and concentration of phytochemical constituents in lotus extracts for cosmetic and food applications remain limited. Therefore, in this study we aimed to develop a safe and biologically active *Nelumbo nucifera* extract suitable for cosmetic, nutraceutical, and pharmaceutical applications. *Nelumbo nucifera* flowers were initially extracted with water, followed by sequential ethanol fractionation. The phytochemical profiles of each fraction were characterized, and their antioxidant and anti-inflammatory activities were assessed via in vitro assays. Selected fractions were then recombined to produce a novel lotus flower extract, which was further evaluated for its antioxidant and anti-inflammatory activities in skin cells, aiming to assess its potential application as an active ingredient in cosmetic products and dietary supplements.

## 2. Materials and Methods

### 2.1. Materials

The chemical standards used for liquid chromatography–electrospray ionization–mass spectrometry (LC-ESI/MS) analysis were sourced from different suppliers. Myricetin, quercetin 3-glucoside, quercetin 3-glucuronide, quercetin 3-galactoside, and kaempferol 3-glucoside were purchased from Sigma-Aldrich (St. Louis, MO, USA). Kaempferol 3-galactoside, kaempferol 3-glucuronide, and isorhamnetin 3-glucoside were obtained from ChemFaces (Wuhan, China). Quercetin 3-rutinoside (rutin) was acquired from Abcam (Cambridge Biomedical Campus, Cambridge, UK). For antioxidant evaluation, 2,2-diphenyl-1-picrylhydrazyl (DPPH), 2,2′-azinobis(3-ethylbenzothiazoline-6-sulfonic acid ammonium salt) (ABTS), and 2,4,6-tris(2-pyridyl)-s-triazine (TPTZ) were purchased from Chemscene (Monmouth Junction, NJ, USA), TCI (Tokyo, Japan), and Sigma-Aldrich (St. Louis, MO, USA), respectively. Sodium acetate, ascorbic acid, gallic acid, potassium persulfate, and iron (III) chloride hexahydrate (FeCl_3_·6H_2_O) were purchased from Sigma-Aldrich (St. Louis, MO, USA). Glacial acetic acid was obtained from Daejung Chemical & Metals (Siheung, Republic of Korea), and iron (II) sulfate heptahydrate (FeSO_4_·7H_2_O) was obtained from Junsei Chemical (Tokyo, Japan).

### 2.2. Preparation of Nelumbo nucifera Flower Water Extract (NFWE), Its Fractions, and Nelumbo nucifera Flower Extract (NFE)

Dried flowers of *Nelumbo nucifera* from the Republic of Korea were purchased from Human Food & Beverage (Gyeongsan, Republic of Korea). The specimen was identified by Dr. Gil Nam Kim from the Oriental Herbal Research Lab, LG Household & Healthcare. A voucher sample (LGHH 24-39) has been deposited at the Oriental Herbal Research Center, LG H&H. Lotus flowers were extracted with distilled water at a weight ratio of 1:20 in a water bath at 80 °C for 3 h. Following cooling at room temperature (20–25 °C), the extract was filtered through Whatman^®^ qualitative filter paper, Grade 4 (Cytiva, Wilmington, DE, USA) to obtain the crude lotus flower water extract (NFWE). A porous resin chromatography column was packed with Diaion^®^ HP-20 (Sigma-Aldrich, St. Louis, MO, USA) resin as the stationary phase. The NFWE was adsorbed onto the column, followed by sequential elution with 100% water and 30%, 70%, and 100% ethanol. Each fraction was concentrated under reduced pressure, and the weight and yield were determined. For the experiments, NFWE fractions were dissolved in dimethylsulfoxide at varying concentrations. The NFE was prepared by dissolving 2.1 g of the 30% ethanol fraction (EF) and 0.7 g of the 70% EF separately in 50% butylene glycol (Duksan Pure Chemicals, Ansan, Republic of Korea) at a weight ratio of 1:100. The two solutions were then combined and the resulting mixture was filtered.

### 2.3. Total Phenolic and Flavonoid Contents

The total phenolic content in the NFWE fractions was determined using a modified Folin–Ciocalteu method [33]. An appropriately diluted 0.1 mL of the NFWE fractions was added to a test tube, followed by 0.8 mL of distilled water. Then, 0.1 mL of Folin–Ciocalteu’s phenol reagent (Sigma-Aldrich, St. Louis, MO, USA) was added and mixed. After a 3–5 min interval, 0.2 mL of saturated 7.5% sodium carbonate (Sigma-Aldrich, St. Louis, MO, USA) solution was added, and the mixture was left to stand for 2 h. Absorbance measurements were taken at 760 nm using a SYNERGY H1 microplate reader (BioTek, Winooski, VT, USA). The total phenolic compound content was quantified as milligrams of gallic acid equivalents (GAE) based on the equation derived from the gallic acid standard calibration curve (equation: y = 0.0012 x − 0.0441, R^2^ = 0.9998).

The total flavonoid levels in the NFWE fractions were quantified using a modified colorimetric assay described by Abeysinghe et al. [34]. A properly diluted 0.1 mL of each NFWE fraction was added to test tubes containing 0.7 mL of absolute ethanol. Subsequently, 0.8 mL of 90% diethylene glycol (Sigma-Aldrich, St. Louis, MO, USA) was added and thoroughly mixed. Following this preparation, 0.1 mL of 1 M sodium hydroxide (Sigma-Aldrich, St. Louis, MO, USA) was added to initiate the reaction. After incubation for 10 min at 40 °C, the absorbance was measured at 420 nm. Rutin was used to establish the standard curve for quantification, and the total flavonoid content was expressed as milligrams of rutin equivalents (RE), calculated from the rutin standard curve equation (equation: y = 0.0008 x − 0.0049, R^2^ = 0.9950).

### 2.4. Liquid Chromatography–Electrospray Ionization/Mass Spectrometry (LC-ESI/MS) Analysis

The reference compound solutions were individually adjusted to a concentration of 5 mg/L by dilution in methanol to serve as reference standards. Each lotus flower fraction (0.3 g) was accurately weighed and then diluted with methanol to a final volume of 100 mL to prepare the sample solution for LC-ESI/MS analysis. Both the sample and the standard solutions were analyzed qualitatively and quantitatively using a liquid chromatography–mass spectrometry (LC/MS) system with electrospray ionization (ESI) (Thermo Scientific, Waltham, MA, USA). Separation was performed using an Agilent Poroshell C18 column (2.1 × 50 mm, particle size 2.7 µm; Agilent, Santa Clara, CA, USA). The mobile phase started with 90% (A) 0.1% formic acid in water and 10% (B) 0.1% formic acid in acetonitrile, followed by the application of a gradient up to 50% (A) and 50% (B) for separation. Qualitative analysis within each fraction was conducted based on retention time, parent ion, and fragment ions, whereas the final quantitative analysis was performed using the parent ion.

### 2.5. DPPH Assay

The DPPH assay was performed in 96-well plates using a SYNERGY H1 microplate reader (BioTek, Winooski, VT, USA), based on the method described by Jing et al. with some modification [35]. DPPH was dissolved in 100% methanol, to yield a stock solution with a final concentration of 1.5 mM. The NFWE fractions, NFE, and the positive control, ascorbic acid, were each serially diluted to appropriate concentrations, and 0.1 mL of each dilution was added to the wells. After the addition of 0.1 mL of 0.15 mM DPPH solution, the plates were incubated for 30 min in the dark at room temperature (20–25 °C). The resultant absorbance at 517 nm was measured. DPPH radical scavenging activity was determined using the following equation:

% inhibition of DPPH activity = [(*A*_DPPH_ − *A*_S_)/*A*_DPPH_] × 100,

In this calculation, *A*_S_ represents the absorbance measured for the sample fractions or extract at a specific concentration, while *A*_DPPH_ designates the baseline absorbance of the DPPH solution in the absence of any sample material. Antioxidant activity was defined by the half-maximal inhibitory concentration (IC_50_), which corresponds to the sample concentration necessary to achieve a 50% reduction in absorbance at 517 nm. The results are expressed in µg/mL or mg/mL. A lower IC_50_ value indicates stronger antioxidant potential.

### 2.6. ABTS Assay

The ABTS radical scavenging activity of the NFWE fractions and NFE was evaluated based on a minor modification of the procedure established by Dhanaraj et al. [36]. Initial stock solutions were prepared, comprising 7 mM ABTS and 2.45 mM potassium persulfate, respectively. The ABTS radical cation solution was prepared by mixing equivalent volumes of these two stock solutions and allowing the mixture to react in the dark at room temperature (20–25 °C) for 17 h. This solution was subsequently diluted with methanol at a ratio of 1:19 (*v*/*v*) to yield the ready-to-use ABTS working solution. Precisely 100 µL of each sample was mixed with 100 μL of the ABTS working solution and incubated for 10 min. The absorbance of the mixture was measured at a wavelength of 734 nm. The antioxidant activity of the NFWE fractions and NFE was evaluated using the IC_50_ value (µg/mL or mg/mL). The percentage inhibition was quantified using the formula presented below:

% inhibition of ABTS activity = [(*A*_ABTS_ − *A*_S_)/*A*_ABTS_] × 100,

*A*_S_ is defined as the absorbance value for the sample fractions or extract at a given concentration, while *A*_ABTS_ represents the absorbance value of the ABTS solution alone.

### 2.7. Ferric Reducing Antioxidant Power (FRAP) Assay

The FRAP assay followed the protocol established by Thaipong et al., with slight modifications [37]. Stock solutions were prepared as follows: 300 mM sodium acetate buffer adjusted to pH 3.6 using glacial acetic acid, 10 mM TPTZ solution dissolved in 40 mM HCl, 20 mM FeCl_3_·6H_2_O solution, and 10 mM FeSO_4_·7H_2_O solution. The working FRAP reagent was freshly prepared by mixing 15 mL of sodium acetate buffer, 1.5 mL of TPTZ solution, and 1.5 mL of FeCl_3_·6H_2_O solution, followed by incubation at 37 °C before use. Aliquots of 100 µL of the NFWE fractions and NFE were mixed with 1900 µL of the FRAP working reagent and incubated for 30 min in the dark. The ferrous tripyridyltriazine complex formed in the reaction was analyzed spectrophotometrically at 593 nm. FRAP values were determined based on a standard curve constructed using various concentrations of FeSO_4_·7H_2_O (equation: y = 0.0012 x − 0.0024, R^2^ = 0.9999).

### 2.8. Cell Culture

RAW264.7 cells, a mouse macrophage line, were obtained from Korean Cell Line Bank (KCLB; Seoul, Republic of Korea). The HaCaT immortalized human keratinocyte line was supplied by AddexBio (San Diego, CA, USA). Both RAW264.7 and HaCaT cells were grown in Dulbecco’s modified Eagle’s medium (DMEM; Solbio, Seoul, Republic of Korea) supplemented with 10% fetal bovine serum (Thermo Fisher Scientific, Waltham, MA, USA) and 1% antibiotic–antimycotic solution (Thermo Fisher Scientific, Waltham, MA, USA). Cultivation was carried out at 37 °C under humidified conditions with 5% carbon dioxide (CO_2_).

### 2.9. Cell Viability Assay

Cell viability was assessed using the cell counting kit-8 assay (CCK-8; Dojindo Molecular Technologies, Mashki, Japan). The RAW264.7 and HaCaT cells were seeded in 96-well plates at densities of 8 × 10^4^ and 1 × 10^5^ cells per well, respectively, and incubated for 24 h. Subsequently, the cells were washed with phosphate-buffered saline (PBS) and treated with various concentrations of NFEW fractions and NFE in serum-free DMEM for 24 h. A 10% CCK-8 reagent was then added to the culture medium, and the cells were incubated for 30 min at 37 °C. The resulting absorbance was determined at 450 nm using a SYNERGY H1 microplate reader (BioTek, Winooski, VT, USA).

### 2.10. Nitric Oxide (NO) Assay

NO levels were quantified using the Griess reagent (Sigma-Aldrich, St. Louis, MO, USA). First, RAW264.7 cells were plated in 24-well plates at a density of 2.5 × 10^5^ cells per well and cultured for 24 h. Serum-free DMEM containing various concentrations of vehicle, NFWE, or NFE was then applied, followed by treatment with 1 μg/mL of lipopolysaccharide (LPS) 1 h later. After the incubation for 24 h, 100 µL of the culture medium was mixed with an equal volume of 40 mg/mL Griess reagent and incubated for 10 min at room temperature (20–25 °C). Absorbance at 540 nm was recorded using a SYNERGY H1 microplate reader (BioTek, Winooski, VT, USA). NO levels were quantified using a nitrite standard curve constructed with sodium nitrite solutions of known concentrations.

### 2.11. RNA Extraction and Real-Time Quantitative Reverse Transcription Polymerase Chain Reaction (qRT-PCR)

HaCaT cells (2 × 10^5^ cells/well) were seeded in 12-well plates and allowed to adhere for 24 h. After washing with PBS, the cells were treated with serum-free DMEM containing either vehicle or NFE for 1 h and subsequently stimulated with tumor necrosis factor-alpha (TNF-α; Sigma-Aldrich) for 24 h. Total RNA was extracted from HaCaT cells using the AccuPrep^®^ Universal RNA Extraction Kit (Bioneer, Daejeon, Republic of Korea) following the manufacturer’s instructions. RNA concentration and purity were determined using a NanoDrop 2000 spectrophotometer (Thermo Fisher Scientific, Waltham, MA, USA). Subsequently, 1 µg of RNA was reverse-transcribed into cDNA using the AccuPower^®^ RocketScript™ RT PreMix & Master Mix (Bioneer, Daejeon, Republic of Korea). Quantitative PCR was carried out on the StepOnePlus^®^ Real-Time PCR System (Applied Biosystems, Waltham, MA, USA) using the following TaqMan probes: interleukin 6 (IL6; Hs00174131_m1), C-X-C motif chemokine ligand 8 (CXCL8; Hs00174103_m1), and glyceraldehyde-3-phosphate dehydrogenase (GAPDH; #4352665; Thermo Fisher Scientific). Relative mRNA expression was calculated using the 2^–∆∆Ct^ method with GAPDH as the internal control.

### 2.12. Enzyme-Linked Immunosorbent Assay (ELISA)

RAW264.7 and HaCaT cells were plated at densities of 2.5 × 10^5^ and 2 × 10^5^ cells per well, respectively, in 24-well plates and incubated for 24 h. After wash with PBS, the cells were treated with either vehicle or NFE in serum-free DMEM for 24 h. Following this incubation, the cell culture supernatants were collected. Levels of prostaglandin E_2_ (PGE_2_)_,_ interleukin (IL)-6, and IL-8 were quantified using the PGE_2_ ELISA kit (Enzo Life Sciences Inc., Farmingdale, NY, USA) and the IL-6 and IL-8 DuoSet ELISA kits (R&D Systems, Minneapolis, MN, USA) in accordance with the manufacturer’s instructions.

### 2.13. Statistical Analysis

Statistical comparisons between two groups were performed using Student’s *t*-test in Microsoft Excel 2016. For comparisons among three or more groups, analysis of variance (ANOVA), followed by Duncan’s multiple range test, was conducted using R software (version 4.5.1). All experiments were carried out in triplicate, and the level of statistical significance was defined as *p* < 0.05.

## 3. Results

### 3.1. Yield and Total Polyphenol and Flavonoid Contents of Nelumbo nucifera Flower Water Extract (NFWE) and Its Fractions

We conducted an aqueous extraction of *Nelumbo nucifera* flower and subsequently fractionated the extract using porous resin column chromatography with solvent systems of 100% water and 30%, 70%, and 100% ethanol. The extraction yields of the NFWE fractions are presented in Table 1. The yield of the water extract was 11.7%.

Polyphenols, natural compounds that consist of two or more phenol structures, are widely found in plants. Flavonoids are a class of polyphenolic compounds with two aromatic rings connected by a heterocyclic ring and are known for diverse biological properties [38]. We found that the fractions derived from the NFWE contained different levels of phenolic and flavonoid compounds. The total polyphenol and flavonoid contents for each of the fractions were analyzed and are described in Table 2. The highest total phenolic content was measured in the 30% EF, followed by 70% EF, 100% EF, and then 100% water extract (WF). The total flavonoid content was highest in the 70% EF, followed by the 30% EF, 100% EF, and 100% WF fractions in descending order.

### 3.2. LC-ESI/MS Analysis of Phytochemicals in NFWE Fractions

**Figure 1:**
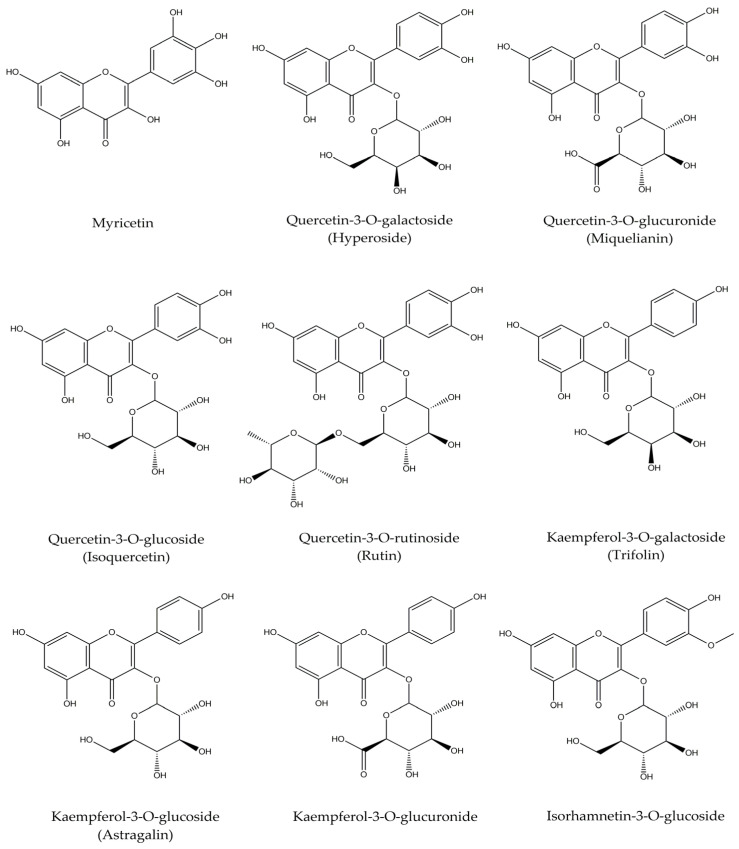
Chemical structures of flavonoids in the NFWE fractions analyzed using LC-ESI/MS. NFWE, *Nelumbo nucifera* flower water extract; LC-ESI/MS, liquid chromatography–electrospray ionization–mass spectrometry.

Numerous studies have reported the presence of diverse bioactive compounds in extracts derived from various parts of the lotus plant [15,19,20,21,22,23,24,25,26,27,28,29,30,31,32]. In this study, to characterize the phytochemical profile of different NFWE fractions, we targeted nine compounds previously identified as major constituents of lotus flowers. These compounds were analyzed using LC-ESI/MS. The chemical structures of the selected compounds are illustrated in Figure 1.

Qualitative identification of each fraction’s constituents was performed based on retention times, parent ions, and fragment ions, whereas quantitative analysis relied on the intensity of parent ion signals (Table 3). The quantification results of these nine compounds across the NFWE fractions are summarized in Table 4. All nine compounds were detected in both the 70% and 100% ethanol fractions (EFs), with the highest overall concentrations found in 70% EF. Among them, myricetin and quercetin-3-O-galactoside exhibited notably high levels, exceeding 100 mg/g. Following these, quercetin 3-glucuronide, kaempferol-3-O-galactoside, kaempferol-3-O-glucoside, and quercetin-3-O-glucoside were present in descending order of abundance. In the 100% EF, myricetin and quercetin-3-O-galactoside remained the most abundant compounds, each detected at concentrations above 10 mg/g, followed by kaempferol-3-O-glucoside, kaempferol-3-O-galactoside, and isorhamnetin-3-O-glucoside.

### 3.3. Antioxidant Activity of NFWE Fractions

Plants contain various bioactive compounds that effectively exert antioxidant functions. In our previous analysis, we confirmed that the fractions derived from NFWE possess notably high levels of polyphenols and flavonoids, which are known contributors to antioxidant activity. To investigate the antioxidant capabilities of these fractions, we employed diverse assays focusing on different mechanisms of antioxidant function. Specifically, the radical scavenging activities of the fractions were evaluated using DPPH and ABTS assays, which quantify their free radical neutralizing capacity. Additionally, the reducing power, which reflects the ability to donate electrons and reduce oxidized intermediates, was assessed through the FRAP assay.

**Figure 2:**
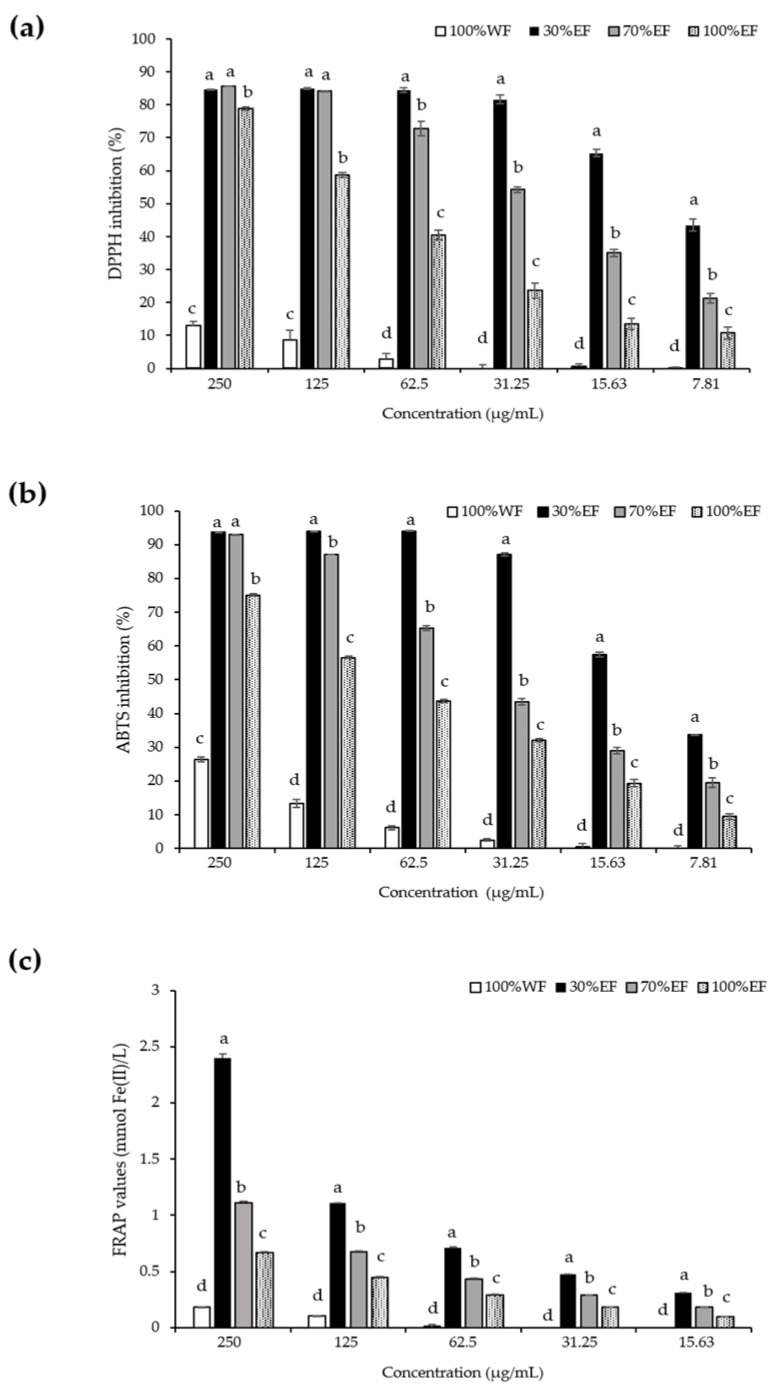
Antioxidant activity of NFWE fractions. The percentages of DPPH (**a**) and ABTS inhibition (**b**) were all referenced relative to the control group. The ferric reducing power (**c**) was represented by FRAP values. All values are represented as mean ± standard deviation from triplicate analyses. Means with different superscripts (a–d) in the fractions indicate significant differences according to one-way analysis of variance (ANOVA), followed by Duncan’s multiple range test at *p* < 0.05. DPPH, 2,2-diphenyl-1-picrylhydrazyl; ABTS, 2,2′-azinobis(3-ethylbenzothiazoline-6-sulfonic acid; FRAP, ferric reducing antioxidant power.

The relative antioxidant activities of NFWE fractions at different concentrations are shown in Figure 2. All fractions displayed an increase in antioxidant activity with increasing concentrations, ranging from 15.63 to 250 μg/mL. Across all assays, the 30% EF consistently demonstrated the highest antioxidant activity, showing strong inhibition of DPPH and ABTS radicals as well as significant ferric reducing capacity. For example, at lower concentrations, the 30% EF maintained over 80% inhibition in both DPPH and ABTS assays, indicating potent free radical scavenging ability. The FRAP assay similarly showed higher reducing power for the 30% EF compared with other fractions. The 70% EF exhibited moderate antioxidant effects, with noticeable but lower radical scavenging and reducing activities than the 30% EF. The 100% EF displayed weaker antioxidant performance, whereas the 100% WF fraction showed the least activity in all assays, with minimal radical inhibition and reducing power even at higher concentrations.

In addition to the antioxidant activity data above, the IC_50_ values for all assays are listed in Table 5. The results confirmed that the 30% EF exhibited the strongest antioxidant activity, approaching that of the standard antioxidant L-ascorbic acid. The 70% EF also showed appreciable antioxidant capacity, though it was less potent than the 30% EF. In contrast, the 100% EF and 100% WF exhibited weaker antioxidant effects, with the 100% WF showing the highest IC_50_ values, indicating the lowest antioxidant activity among the fractions tested. Overall, these results suggest that the 30% EF possesses the most potent antioxidant properties among the fractions derived from the NFWE, closely followed by the 70% EF.

### 3.4. Inhibitory Effect of NFWE Fractions on NO Production in RAW264.7 Cells

**Figure 3:**
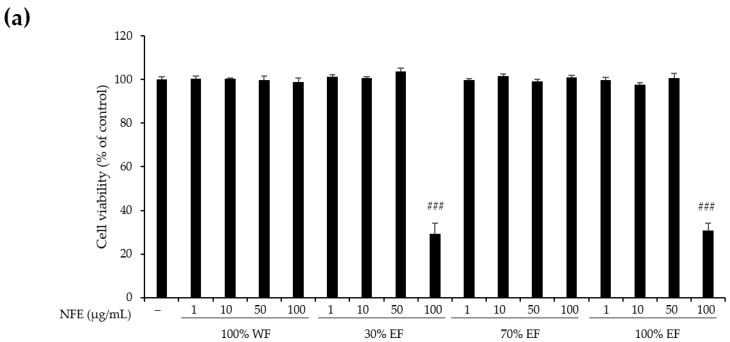

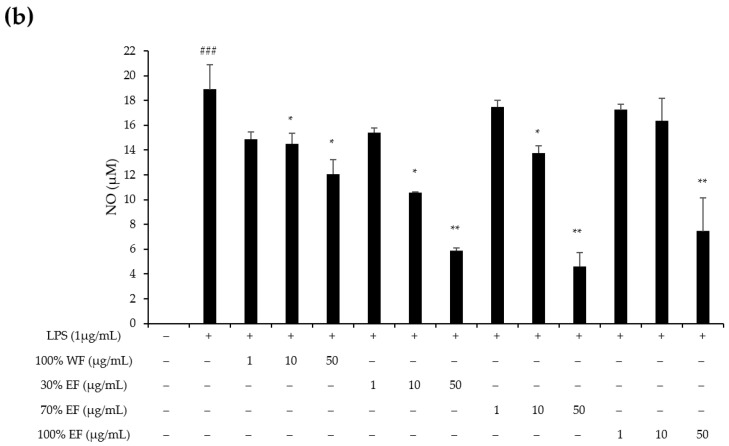
Effect of NFWE fractions on NO production in the LPS-induced RAW264.7 cells. (**a**) The RAW264.7 cells were treated with NFWE fractions at different concentrations (1, 10, 50, and 100 μg/mL) for 24 h. Cell viability was assessed using the CCK-8 assay. The values are expressed as means ± standard deviation. ### *p* < 0.001, compared with the control group. (**b**) RAW264.7 cells were pretreated with the specified concentrations of NFWE fractions for 1 h before being exposed to LPS (1 μg/mL) for 24 h. NO production was then measured using the Griess reaction assay. The values are expressed as mean ± standard deviation. Statistical comparisons between groups were conducted using Student’s *t*-test. ### *p* < 0.001, compared with the control group. * *p* < 0.05, ** *p* < 0.01 compared with the LPS group. NO, nitric oxide; LPS, lipopolysaccharide; CCK-8, cell counting kit-8.

Plant extracts with antioxidant properties are known to possess various biological activities such as anti-inflammatory, antiviral, and antibacterial effects [39,40,41]. To determine whether the NFWE fractions with strong antioxidant activity also have anti-inflammatory effects, we examined their ability to inhibit LPS-induced NO production in RAW264.7 cells. To identify the concentrations of each fraction that do not exhibit toxicity to RAW264.7 cells, the cells were treated with varying doses of each fraction (1, 10, 50, and 100 μg/mL) and cell viability was evaluated using the CCK-8 assay (Figure 3a). Since 30% EF and 100% EF were toxic to RAW264.7 cells at 100 µg/mL, the NO assay was conducted at concentrations ranging from 1 to 50 µg/mL. As shown in Figure 3b, all the NFWE fractions caused dose-dependent inhibition of NO production induced by LPS in RAW264.7 cells. Notably, the 30% EF and 70% EF exhibited particularly strong inhibition of NO generation. This suggests that the NFWE fractions effectively reduce the inflammatory response triggered by LPS in macrophages, highlighting their potential anti-inflammatory properties.

### 3.5. Antioxidant Effect of Nelumbo nucifera Flower Extract (NFE)

**Figure 4:**
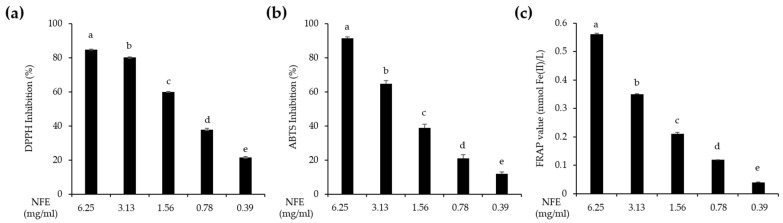
Antioxidant activity of NFE. The percentages of DPPH (**a**) and ABTS inhibition (**b**) were all referenced relative to the control group. The ferric reducing power (**c**) was represented by FRAP values. All values are represented as mean ± standard deviation from triplicate analyses. Means with different superscripts (a–e) in the fractions indicate significant differences according to one-way analysis of variance, followed by Duncan’s multiple range test at *p* < 0.05. NFE, *Nelumbo nucifera* flower extract; DPPH, 2,2-diphenyl-1-picrylhydrazyl; ABTS, 2,2′-azinobis(3-ethylbenzothiazoline-6-sulfonic acid; FRAP, ferric reducing antioxidant power.

Earlier findings in this study revealed that the 30% EF and 70% EF of NFWE contained higher levels of polyphenols and flavonoids compared with other fractions and exhibited greater antioxidant and anti-inflammatory activities. Based on these results, we combined these two fractions to create a single extract, NFE, which includes a diverse spectrum of polyphenolic constituents with the aim of enhancing overall biological activity. The antioxidant activity of the extract was measured, and the findings are presented in Table 6 and Figure 4.

The IC_50_ values of NFE were 0.98 mg/mL for DPPH, 2.02 mg/mL for ABTS, and 19.25 mg/mL for the FRAP assay. In comparison, the standard antioxidant L-ascorbic acid exhibited lower IC_50_ values of 5.84 ± 0.45 μg/mL, 9.63 ± 0.20 μg/mL, and 52.03 ± 2.51 μg/mL for the same assays, respectively (Table 6). Although the IC_50_ values of NFE were higher than those of the L-ascorbic acid on a weight basis, indicating relatively lower potency, NFE still demonstrated considerable antioxidant efficacy (Figure 4). Both the DPPH and ABTS assays exhibited a clear dose-dependent increase in radical scavenging activity with increasing NFE concentrations. At the highest concentration tested (6.25 mg/mL), NFE showed the strongest free radical inhibition, indicating potent antioxidant effects. Similarly, FRAP values increased with rising NFE concentrations, reflecting enhanced ferric ion (Fe^3+^) reducing power. These results indicate that NFE possesses strong, concentration-dependent antioxidant activities.

### 3.6. Anti-Inflammatory Activities of NFE in RAW264.7 and HaCaT Cells

**Figure 5:**
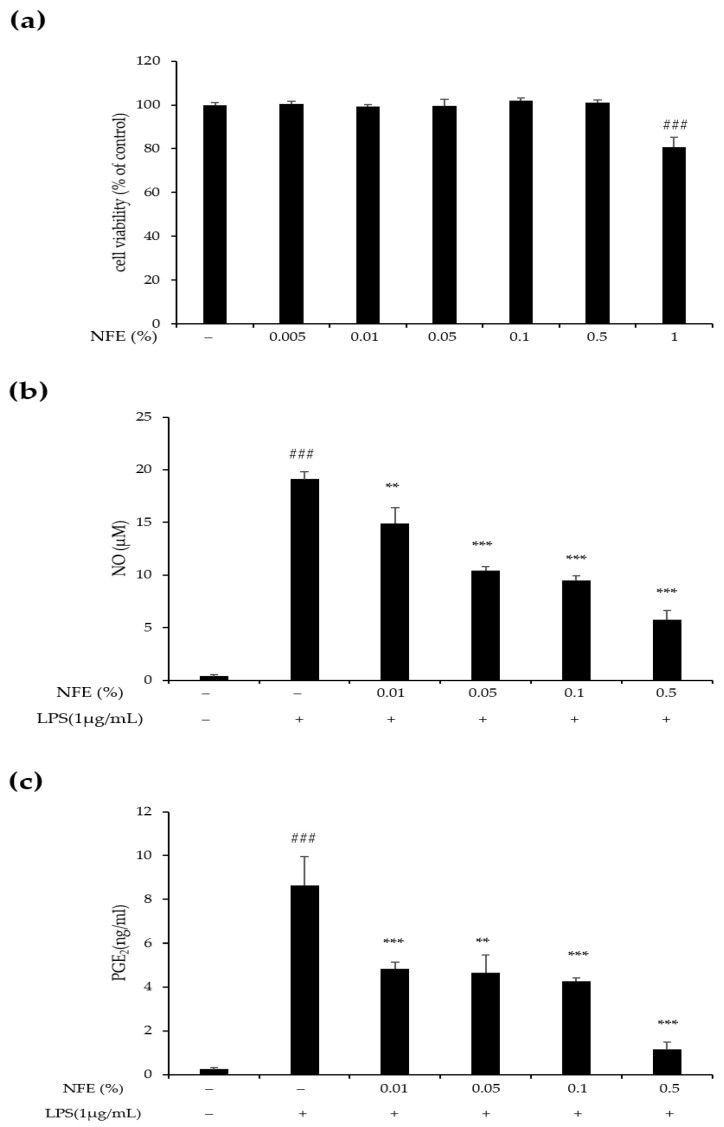
Inhibitory effect of NFE on NO and PGE_2_ production in the LPS-activated RAW264.7 cells. (**a**) RAW264.7 cells were exposed to NFE at varying concentrations (0.005, 0.01, 0.05, 0.1, 0.5, and 1%) for 24 h. Cell viability was assessed using the CCK-8 assay. (**b**) RAW264.7 cells were pretreated with the specified concentrations (0.01, 0.05, 0.1, and 0.5%) of NFE for 1 h prior to being subjected to LPS (1 μg/mL) for 24 h. NO production was then quantified using the Griess reaction assay. (**c**) PGE_2_ levels in the cell culture supernatant were measured by ELISA. The values are expressed as mean ± standard deviation. ### *p* < 0.001, compared with the control group. ** *p* < 0.01, and *** *p* < 0.001 compared with the LPS treated group. PGE_2,_ prostaglandin E_2_; ELISA, enzyme-linked immunosorbent assay; NFE, *Nelumbo nucifera* flower extract; NO, nitric oxide; LPS, lipopolysaccharide.

Before evaluating the anti-inflammatory effects of NFE, the non-cytotoxic concentration of NFE on RAW264.7 cells was determined using a CCK-8 assay. The results demonstrated that NFE did not affect cell viability at concentrations up to 0.5% (Figure 5a). The anti-inflammatory activity of NFE was evaluated by measuring the suppression of NO and PGE_2_ production in LPS-stimulated RAW264.7 cells. As shown in Figure 5b, pretreatment with NFE led to a concentration-dependent reduction in NO levels elevated by LPS. Similarly, LPS-induced PGE_2_ production was significantly inhibited by NFE in a dose-dependent manner (Figure 5c). These results indicate that NFE effectively reduces the production of the inflammatory mediators NO and PGE_2_, confirming its anti-inflammatory properties.

**Figure 6:**
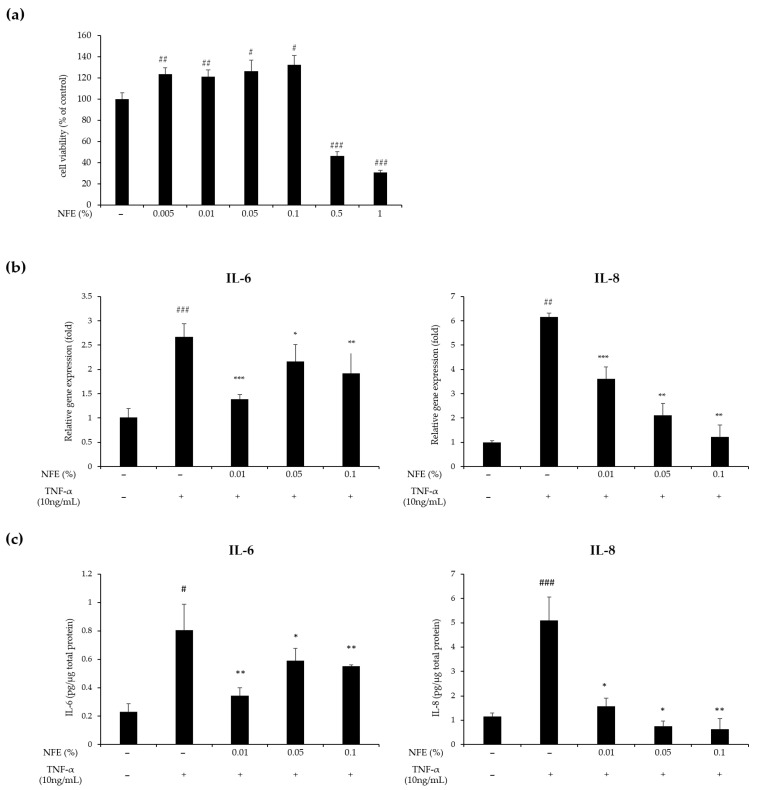
Anti-inflammatory effects of NFE in HaCaT cells. (**a**) Cell viability of HaCaT cells treated with NFE at concentrations ranging from 0.005% to 0.1% for 24 h was measured using the CCK-8 assay. (**b**) HaCaT cells were pretreated with NEF at the indicated concentrations (0.01, 0.05, and 0.1%) and subsequently stimulated with TNF-α for 24 h. mRNA expression IL-6 and IL-8 was determined using qPCR. (**c**) Protein concentrations of IL-6 and IL-8 in HaCaT cells were assessed by ELISA and normalized to the total protein content. Data are expressed mean ± standard deviation of three independent experiments. Statistical significance is denoted as # *p* < 0.05, ## *p* < 0.01, and ### *p* < 0.001 versus the control group; * *p* < 0.05, ** *p* < 0.01, and *** *p* < 0.001 versus the TNF-α treated group. TNF, tumor necrosis factor; IL, interleukin; qPCR, quantitative polymerase chain reaction; NFE, *Nelumbo nucifera* flower extract.

We also confirmed the anti-inflammatory effects of NFE in HaCaT cells, an immortalized human keratinocyte cell line. Using the CCK-8 assay, we found that NFE at concentrations up to 0.1% did not affect cell viability, indicating no cytotoxicity within this range (Figure 6a). HaCaT cells were pretreated with NFE at 0.01%, 0.05%, and 0.1% and then stimulated with TNF-α to induce an inflammatory response. Quantitative real-time PCR (qPCR) analysis showed that TNF-α significantly increased mRNA expression of the inflammatory cytokines IL-6 and IL-8. However, pretreatment with NFE significantly reduced these elevated expressions. Notably, IL-8 mRNA expression was decreased in a dose-dependent manner by NFE. Furthermore, ELISAs demonstrated that NFE also significantly decreased the TNF-α-induced protein levels of IL-6 and IL-8, following a pattern similar to the mRNA results. Particularly, IL-8 protein levels were dramatically reduced at all tested concentrations. These findings suggest that NFE effectively modulates inflammatory cytokine expression in skin cells, thereby exerting anti-inflammatory effects.

## 4. Discussion

In this study, we quantified the total polyphenol and flavonoid contents, as well as various phytochemicals, in different fractions of NFWE. We also evaluated the antioxidant and anti-inflammatory activities of each fraction. Notably, the 30% and 70% EFs demonstrated superior bioactivities, leading to their combination to formulate a novel extract, designated as NFE. Subsequent analyses revealed that NFE exhibited remarkably potent antioxidant and anti-inflammatory activities.

Numerous studies have demonstrated that extracts from various parts of *Nelumbo nucifera* contain abundant polyphenolic compounds, including flavonoids and phenolic acids, and that these constituents contribute to the plant’s broad biological activities [15,19,20,21,22,23,24,25,26,27,28,29,30,31,32]. Furthermore, *Nelumbo nucifera* flowers contain higher levels of total polyphenols than other parts such as leaves, rhizomes, and seeds, correlating with superior antioxidant efficacy [42]. Lotus flowers exhibit relatively lower biomass compared with other plant organs such as rhizomes and leaves. Nevertheless, harvesting flowers inflicts less damage on the overall plant structure and function than the collection of other parts. Harvesting rhizomes or leaves often necessitates uprooting or damaging essential structural components, which can adversely affect plant survival and growth. In contrast, flower harvesting is a less invasive practice that minimally disrupts the plant’s physiological processes and persistence. Consequently, prioritizing flower harvest may represent a more sustainable strategy for resource utilization, enabling the acquisition of valuable raw materials while mitigating negative impacts on plant populations. Given these considerations, we selected the flowers among the various parts of the *Nelumbo nucifera* for extraction and subsequent evaluation in this study.

Safety considerations were prioritized when selecting the extraction solvent. Although organic solvents such as methanol, butanol, hexane, chloroform, or ethyl acetate are well recognized for their superior extraction efficiency and ability to isolate bioactive compounds, their use in cosmetic and food applications is constrained by potential toxicity and regulatory limitations. Water is a non-toxic, biocompatible solvent widely regarded as the safest choice in the food and cosmetic industries. It is particularly suitable for the extraction of polar and moderately polar compounds. Although polyphenols and flavonoids are commonly described as hydrophobic, they are not strictly nonpolar; their solubility varies depending on their molecular structure and extraction parameters. Flavonoids containing hydroxyl (–OH) groups impart considerable polarity, which enhances their solubility in polar solvent and aqueous media [43]. Within plant tissues, these compounds often occur as glycosides—conjugated with sugar moieties—which further enhances their aqueous solubility [44]. Additionally, the application of elevated temperatures during water-based extraction can significantly improve extraction efficiency and increase polyphenol yield [45]. Therefore, aqueous extraction using hot water was employed to obtain the lotus flower extract, ensuring a safer and more biocompatible process.

To selectively separate and enrich various compounds within NFWE, stepwise fractionation using ethanol at varying concentrations was conducted based on polarity differences. The yield of each fraction was carefully evaluated. The hot water extract was fractionated, resulting in a predominance of highly polar water-soluble components, which led to the highest yield in the 100% water fraction. As the ethanol concentration increased, the types of soluble compounds became more limited, causing the yield to decrease progressively from 30% to 70% and 100% EFs (Table 1). Quantitative analysis of polyphenol and flavonoid contents within each fraction revealed that polyphenol levels were highest in the 30% EF, followed by the 70%, 100% EFs, and 100% WF, respectively. Conversely, flavonoid content was most abundant in the 70% EF, followed by the 30%, 100% EFs, and 100% WF (Table 2). This difference in total polyphenol and flavonoid contents is likely due to the differing polarity of the 30% and 70% ethanol solvents. These findings suggest that the polyphenolic compounds present in lotus flowers possess relatively higher polarity compared to flavonoids.

To identify the components within each fraction, nine representative phytochemicals known to be prevalent in lotus flower extracts were analyzed via LC-ESI/MS (Table 4). Notably, none of the targeted phytochemicals were detected in the 100% WF, which is likely attributable to the high presence of water-soluble macromolecules such as polysaccharides, proteins, and minerals, coupled with a markedly low flavonoid concentration. In contrast, phytochemicals commonly reported as abundant in lotus flower extracts were predominantly identified in the 70% and 100% EFs. This observation is closely related to the fact that previous studies have typically employed methanol or ethanol as extraction solvents [15,31]. Both methanol and ethanol exhibit relatively lower polarity compared with the solvent system used in the present study, which can significantly influence the solubility and extraction efficiency of various phytochemicals. Specifically, solvents with lower polarity tend to extract flavonoids and other moderately polar to nonpolar compounds more effectively. Therefore, the higher concentration of phytochemicals observed in the 70% and 100% ethanol fractions likely arises from differences in solvent polarity affecting extraction efficiency. Additionally, the nine flavonoids analyzed in this study are also known to be in lotus leaf extracts [29,30]. In particular, a study by Lee et al. analyzed water extracts of lotus leaves collected from various regions in South Korea and compared the levels of flavonoids such as myricetin, isorhamnetin 3-O-glucoside, quercetin, kaempferol 3-O-glucoside, quercetin 3-O-glucoside, rutin, and quercetin 3-O-glucuronide [30]. Although the concentrations varied by region, all of these compounds were detected in every lotus leaf extract, with quercetin 3-O-glucuronide being the most abundant. These compounds were also found in the NFWE fractions, where myricetin was the predominant flavonoid.

The radical scavenging activities of each NFWE fraction were evaluated using DPPH and ABTS assays, whereas reducing antioxidant capacity was assessed via the FRAP assay. The antioxidant activities of these fractions were observed to decrease in the following order: 30% EF > 70% EF > 100% EF > 100% WF (Figure 2, Table 5). This trend exhibited a robust positive correlation with the total polyphenolic content across the fractions. These findings corroborate previous reports demonstrating a significant association between the polyphenol concentration in lotus flower extracts and their potent antioxidant properties, characterized by effective free radical neutralization [42].

The antioxidant potential of plant extracts largely stems from their ability to scavenge ROS, which subsequently suppresses inflammatory signaling pathways, thereby conferring anti-inflammatory effects [39,41]. Accordingly, we investigated the anti-inflammatory activity of the NFWE fractions. The anti-inflammatory efficacy of NFWE fractions was evaluated using the widely employed NO assay, which measures the amount of NO produced in LPS-stimulated RAW264.7 macrophages to assess the inflammatory response. All NFWE fractions inhibited LPS-induced NO production in a concentration-dependent manner, with the 30% and 70% EFs exhibiting markedly superior anti-inflammatory efficacy (Figure 3). Previous research has demonstrated that ethyl acetate and ethanol extracts of lotus flower significantly inhibit the production of TNF-α, a key pro-inflammatory cytokine released by LPS-activated human macrophages, thereby exerting anti-inflammatory effects. Although the extraction methods differ from those employed in the present study, these findings are consistent with our observation that NFWE fractions suppress LPS-induced inflammatory responses in murine macrophages, supporting their anti-inflammatory potential.

The 30% and 70% EFs, characterized by high flavonoid content and potent antioxidant and anti-inflammatory activities, were combined to produce a novel extract, *Nelumbo nucifera* flower extract (NFE). These two fractions exhibit distinct polarity profiles, resulting in complementary phytochemical compositions. This study differs from previous research in that the *Nelumbo nucifera* flower extract was systematically fractionated, and the total polyphenol and flavonoid contents, along with key phytochemical components, were analyzed for each fraction. The combination of fractions with different polarity profiles represents a novel approach that allows for a broader range of bioactive compounds to be included, potentially enhancing the overall biological efficacy compared with individual fractions. Additionally, this approach can reduce processing steps during production when applied as raw materials in cosmetics or food products, thereby saving time and costs. The antioxidant capacity of the resulting NFE was also evaluated and found to be comparable to those of the individual fractions (Table 6, Figure 4). Although NFE may appear to exhibit inferior antioxidant activity relative to L-ascorbic acid, this is primarily due to the dilution and blending of its constituent fractions during formulation. To further evaluate its anti-inflammatory potential, we employed the RAW264.7 murine macrophage cell line. NFE demonstrated a significant, concentration-dependent suppression of key inflammatory mediators, including NO and PGE_2_, both of which are upregulated upon LPS stimulation (Figure 5). Additionally, the anti-inflammatory effects of NFE were confirmed in skin cells. In HaCaT keratinocytes, NFE markedly reduced both the mRNA expression and protein secretion of pro-inflammatory cytokines IL-6 and IL-8, which are upregulated by TNF-α treatment (Figure 6). These findings indicate that NFE effectively attenuates inflammatory responses in macrophages and epidermal skin cells. Therefore, the findings of this study demonstrate that NFE possesses potent antioxidant and anti-inflammatory activities while being safe for potential application as an ingredient in cosmetic or functional food formulations.

Numerous studies have documented the diverse dermatological benefits of lotus extracts, including antioxidant, anti-inflammatory, whitening, and anti-aging properties. Among these, the efficacy of lotus flower extracts on skin health has also been reported. Tungmunnithum et al. demonstrated that *Nelumbo nucifera* flower extracts, particularly stamen extracts, inhibited tyrosinase and collagenase activities in vitro, indicating their potential role in skin aging prevention and whitening enhancement [15]. Zheng et al. also reported that lotus stamen extracts inhibited glycation and cellular senescence induced by high glucose in human fibroblasts [19]. It has been reported that flavonoid compounds, which are abundantly present in lotus extracts, exhibit multiple beneficial effects on the skin. In particular, one study reported an in silico molecular docking analysis of seven major flavonoid compounds extracted from *Nelumbo nucifera*—including myricetin 3-glucoside, quercetin 3-rutinoside, quercetin 3-glucuronide, kaempferol 3-robinobioside, kaempferol 3-glucoside, kaempferol 3-glucuronide, and isorhamnetin 3-glucoside—against three skin aging-related enzymes (collagenase, elastase, and tyrosinase) to evaluate their potential anti-aging effects [46]. Myricetin, a major component of NFE, reportedly inhibits UV-induced photoaging, alleviates inflammation, and ameliorates atopic dermatitis [47,48,49,50,51,52]. Another key constituent, quercetin 3-glucuronide, has also demonstrated antioxidant, anti-inflammatory, moisturizing, and whitening effects in skin cells [53]. Considering these research findings collectively, the NFE examined in this research holds potential for a broad spectrum of beneficial dermatological effects beyond its antioxidant and anti-inflammatory activities. Furthermore, these results suggest that the NFE possesses considerable value as a functional ingredient for applications in cosmetics, nutraceuticals, and pharmaceuticals.

Although NFE exhibits pronounced antioxidant and anti-inflammatory activities and shows promising dermatological potential, several limitations remain to be addressed. First, the molecular mechanisms mediating the antioxidant and anti-inflammatory effects of NFE remain to be fully elucidated. The antioxidant activity is likely mediated through activation of the Nrf2/ARE pathway, which regulates the expression of various cytoprotective genes, such as heme oxygenase-1 (HO-1), NAD(P)H quinone dehydrogenase 1 (NQO1), and glutathione S-transferase (GST) [54]. Meanwhile, the anti-inflammatory effects may involve modulation of key inflammatory mediators such as inducible nitric oxide synthase, cyclooxygenase-2, and the nuclear factor-kappa B signaling pathway [55,56]. Detailed studies focusing on these specific signaling pathways and gene expression profiles involved in both antioxidant responses and inflammation modulation are needed to better understand the mode of action of NFE. Second, although in vitro assays provided valuable insights into the skin-related bioactivities of NFE, in vivo studies and clinical evaluations are essential to confirm its efficacy and safety in real-world applications. Future research should include 3D skin models and human trials to validate the therapeutic potential of NFE for skin health. Lastly, exploring additional skin benefits such as wound healing, UV protection, and barrier function enhancement could expand the applicability of NFE in cosmetic and pharmaceutical formulations. Addressing these aspects will provide a more comprehensive understanding of the capabilities of NFE and support its development as a multifunctional ingredient.

## 5. Conclusions

In this study, we successfully characterized the phytochemical profiles and biological activities of different fractions derived from *Nelumbo nucifera* flower water extract. The 30% and 70% ethanol fractions exhibited the highest total polyphenol and flavonoid contents, with major flavonoids such as myricetin and quercetin derivatives identified by LC-ESI/MS analysis. By combining these two fractions, we developed a novel NFE that demonstrated potent antioxidant activity across multiple assays, including DPPH, ABTS, and FRAP.

Importantly, NFE showed significant anti-inflammatory effects in vitro by markedly inhibiting the production of key inflammatory mediators such as NO and PGE_2_ in LPS-stimulated RAW264.7 macrophages. Furthermore, in human keratinocyte HaCaT cells, NFE effectively reduced TNF-α-induced expression and secretion of pro-inflammatory cytokines IL-6 and IL-8 without cytotoxic effects. These results indicate that NFE can simultaneously mitigate oxidative stress and inflammation, two major contributors to skin aging and inflammatory skin conditions.

The use of lotus flowers as a raw material offers a sustainable advantage over other plant parts, as flower harvesting causes less damage to the plant while providing rich bioactive compounds. Additionally, our extraction method using hot water followed by stepwise ethanol fractionation ensures a safe and biocompatible process suitable for cosmetic and nutraceutical applications.

Despite the promising in vitro results, further studies are needed to elucidate the molecular mechanisms underlying the antioxidant and anti-inflammatory effects of NFE, particularly focusing on signaling pathways such as Nrf2/ARE and NF-κB. Moreover, in vivo evaluations using 3D skin models and clinical trials are essential to confirm the efficacy and safety of NFE for real-world skin health applications. Exploring additional benefits such as UV protection, wound healing, and skin barrier enhancement could further expand the potential uses of this extract.

Overall, NFE represents a valuable natural resource with multifunctional properties that support its development as an active ingredient in cosmetic, nutraceutical, and pharmaceutical products aimed at improving skin health and combating aging-related oxidative and inflammatory damage.

## Figures and Tables

**Table 1:** Extraction yields of the NFWE fractions.

Fractions	Yield (%)
100% WF	11.29
30% EF	2.93
70% EF	0.96
100% EF	0.05

The values listed in the table are expressed as percentages by weight (*w*/*w*) relative to the total weight of the raw material (100%). NFWE, *Nelumbo nucifera* flower water extract; WF, water fraction; EF, ethanol fraction.

**Table 2:** Total polyphenol and flavonoid contents in the fractions obtained from NFWE.

Fractions	Total Phenolic Content (mg GAE/g of DE)	Total Flavonoid Content (mg RE/g of DE)
100% WF	14.12 ± 0.12 ^d^	6.81 ± 0.63 ^c^
30% EF	137.45 ± 2.65 ^a^	102.85 ± 29.19 ^b^
70% EF	63.98 ± 3.61 ^b^	440.15 ± 41.08 ^a^
100% EF	49.26 ± 6.29 ^b^	100.15 ± 10.76 ^b^

All values are represented as mean ± standard deviation from triplicate analyses. Means with different superscripts (^a–d^) in the fractions indicate significant differences according to one-way analysis of variance (ANOVA), followed by Duncan’s multiple range test at *p* < 0.05. GAE, gallic acid equivalents; DE, dry extract; RE, rutin equivalents; WF, water fraction; EF, ethanol fraction.

**Table 3:** LC-MS data of flavonoids derived from NFWE fractions using positive and negative ionization modes.

Flavonoids	Retention Time (Rt)	Fragment Ion (*m*/*z*)	ESI Mode	Parent Ion (*m*/*z*)
Myricetin	3.72	151.616	Negative	316.977
Quercetin-3-O-galactoside (Hyperoside)	3.49	300.424	Positive	463.170
Quercetin-3-O-glucuronide (Miquelianin)	3.40	301.466	Positive	477.100
Quercetin-3-O-glucoside (Isoquercetin)	3.42	300.424	Positive	462.951
Quercetin-3-O-rutinoside (Rutin)	3.33	271.486	Positive	609.062
Kaempferol-3-O-galactoside (Trifolin)	3.65	287.449	Negative	449.125
Kaempferol-3-O-glucoside (Astragalin)	3.70	314.403	Positive	477.052
Kaempferol-3-O-glucuronide	3.66	309.355	Negative	471.117
Isorhamnetin-3-O-glucoside	3.66	228.124	Positive	447.053

ESI, electrospray ionization; NFWE, *Nelumbo nucifera* flower water extract; LC-MS, liquid chromatography–mass spectrometry.

**Table 4:** Concentration (mg/g of dry NFWE) of selected phytochemicals in the NFWE fractions as determined by LC-ESI/MS analysis.

Flavonoids	Concentration (mg/g of Dry NFWE)			
	100% WF	30% EF	70% EF	100% EF
Myricetin	_^1^	_	133.70 ± 6.41 ^a^	13.47 ± 0.93 ^b^
Quercetin-3-O-galactoside (Hyperoside)	_	_	104.53 ± 3.16 ^a^	11.1 ± 1.25 ^b^
Quercetin-3-O-glucuronide (Miquelianin)	_	5.06 ± 0.27 ^b^	62.43 ± 1.35 ^a^	1.50 ± 0.07 ^c^
Quercetin-3-O-glucoside (Isoquercetin)	_	0.03 ± 0.00 ^c^	15.70 ± 0.72 ^a^	1.53 ± 0.10 ^b^
Quercetin-3-O-rutinoside (Rutin)	_	0.20 ± 0.01 ^b^	9.05 ± 0.35 ^a^	0.20 ± 0.02 ^b^
Kaempferol-3-O-galactoside (Trifolin)	_	_	40.17 ± 1.91 ^a^	2.00 ± 0.14 ^b^
Kaempferol-3-O-glucoside (Astragalin)	_	_	34.47 ± 0.85 ^a^	5.62 ± 0.13 ^b^
Kaempferol-3-O-glucuronide	_	_	2.34 ± 0.09 ^a^	0.50 ± 0.03 ^b^
Isorhamnetin-3-O-glucoside	_	0.02 ± 0.00 ^c^	8.86 ± 0.25 ^a^	1.74 ± 0.19 ^b^

_^1^ not detected in the samples. Limit of Quantitation: 0.01 mg/g. All values are represented as mean ± standard deviation from triplicate analyses. Means with different superscripts (^a–c^) in the fractions indicate significant differences according to one-way analysis of variance (ANOVA), followed by Duncan’s multiple range test at *p* < 0.05. LC-ESI/MS, liquid chromatography–electrospray ionization–mass spectrometry; NFWE, *Nelumbo nucifera* flower water extract; WF, water fraction; EF, ethanol fraction.

**Table 5:** Antioxidant activities of various fractions obtained from NFWE.

Fractions	IC_50_ (µg/mL)		
	DPPH	ABTS	FRAP
100% WF	402.02 ± 14.90 ^a^	721.43 ± 12.17 ^a^	3577.68 ± 148.05 ^a^
30% EF	8.20 ± 0.50 ^c^	11.70± 0.15 ^c^	140.11 ± 1.08 ^c^
70% EF	21.50 ± 1.22 ^c^	37.04 ± 0.62 ^c^	425.28 ± 22.12 ^c^
100% EF	67.43 ± 2.50 ^b^	88.90 ± 2.82 ^b^	1058.07 ± 90.35 ^b^
L-ascorbic acid	5.60 ± 0.39	9.11 ± 0.00	52.05 ± 2.51

All values are represented as mean ± standard deviation from triplicate analyses. Means with different superscripts (^a–c^) in the fractions indicate significant differences according to one-way analysis of variance, followed by Duncan’s multiple range test at *p* < 0.05. IC_50_, half-maximal inhibitory concentration; DPPH, 2,2-diphenyl-1-picrylhydrazyl; ABTS, 2,2′-azinobis(3-ethylbenzothiazoline-6-sulfonic acid; FRAP, ferric reducing antioxidant power.

**Table 6:** IC_50_ values of NFE obtained from 30% EF and 70% EF of NFWE.

	IC_50_		
	DPPH	ABTS	FRAP
NFE (mg/mL)	0.98 ± 0.01	2.02 ± 0.10	19.25 ± 0.88
L-ascorbic acid (μg/mL)	5.84 ± 0.45	9.63 ± 0.20	52.03 ± 2.51

All values are represented as mean ± standard deviation from triplicate analyses. IC_50_, half-maximal inhibitory concentration; NFE, *Nelumbo nucifera* flower extract; NFWE, *Nelumbo nucifera* flower water extract; DPPH, 2,2-diphenyl-1-picrylhydrazyl; ABTS, 2,2′-azinobis(3-ethylbenzothiazoline-6-sulfonic acid; FRAP, ferric reducing antioxidant power.

## Data Availability

The datasets supporting the results of the current study are included in this article.

## Abbreviations

The following abbreviations are used in this manuscript:

NFWE	*Nelumbo nucifera* flower water extract
NFE	*Nelumbo nucifera* flower extract
NO	nitric oxide
PGE_2_	prostaglandin E_2_
LPS	Lipopolysaccharide
UV	Ultraviolet
ROS	reactive oxygen species
MMP	matrix metalloproteinase
IL	interleukin
TNF-α	tumor necrosis factor-α
WF	water fraction
EF	ethanol fraction
GAE	gallic acid equivalents
DE	dry extract
RE	rutin equivalents
DPPH	2,2-diphenyl-1-picrylhydrazyl
ABTS	2,2′-azinobis(3-ethylbenzothiazoline-6-sulfonic acid)
TPTZ	2,4,6-tris(2-pyridyl)-s-triazine
FRAP	Ferric Reducing Antioxidant Power
qPCR	quantitative real-time polymerase chain reaction
ELISA	enzyme-linked immunosorbent assay
ANOVA	analysis of variance

## References

[1] Ageing and Health. https://www.who.int/news-room/fact-sheets/detail/ageing-and-health.

[2] Guido G., Ugolini M.M., Sestino A. (2022). Active ageing of elderly consumers: Insights and opportunities for future business strategies. SN Bus. Econ..

[3] Ganceviciene R., Liakou A.I., Theodoridis A., Makrantonaki E., Zouboulis C.C. (2012). Skin anti-aging strategies. Derm.-Endocrinol..

[4] Rinnerthaler M., Bischof J., Streubel M.K., Trost A., Richter K. (2015). Oxidative stress in aging human skin. Biomolecules.

[5] Shin S.H., Lee Y.H., Rho N.-K., Park K.Y. (2023). Skin aging from mechanisms to interventions: Focusing on dermal aging. Front. Physiol..

[6] Agrawal R., Hu A., Bollag W.B. (2023). The skin and Inflamm-aging. Biology.

[7] Salminen A., Kaarniranta K., Kauppinen A. (2022). Photoaging: UV radiation-induced inflammation and immunosuppression accelerate the aging process in the skin. Inflamm. Res..

[8] Michalak M. (2023). Plant extracts as skin care and therapeutic agents. Int. J. Mol. Sci..

[9] Michalak M. (2022). Plant-derived antioxidants: Significance in skin health and the ageing process. Int. J. Mol. Sci..

[10] Chen S., Wu B.-H., Fang J.-B., Liu Y.-L., Zhang H.-H., Fang L.-C., Guan L., Li S.-H. (2012). Analysis of flavonoids from lotus (*Nelumbo nucifera*) leaves using high performance liquid chromatography/photodiode array detector tandem electrospray ionization mass spectrometry and an extraction method optimized by orthogonal design. J. Chromatogr. A.

[11] Tungmunnithum D., Pinthong D., Hano C. (2018). Flavonoids from *Nelumbo nucifera* Gaertn., a medicinal plant: Uses in traditional medicine, phytochemistry and pharmacological activities. Medicines.

[12] Lee H.K., Choi Y.M., Noh D.O., Suh H.J. (2005). Antioxidant effect of Korean traditional lotus liquor (Yunyupju). Int. J. Food Sci. Technol..

[13] Sheikh S.A. (2014). Ethno-medicinal uses and pharmacological activities of lotus (*Nelumbo nucifera*). J. Med. Plants Stud..

[14] Paudel K.R., Panth N. (2015). Phytochemical Profile and Biological Activity of *Nelumbo nucifera*. Evid. Based Complement. Altern. Med..

[15] Tungmunnithum D., Drouet S., Hano C. (2022). Validation of a high-performance liquid chromatography with photodiode array detection method for the separation and quantification of antioxidant and skin anti-aging flavonoids from *Nelumbo nucifera* Gaertn. Stamen extract. Molecules.

[16] Iwamoto A., Yamauchi R., Oogai S., Tsuruta Y., Keisuke T., Nagata Y., Yanagita T. (2022). Lotus root extract inhibits skin damage through suppression of collagenase production in vitro. Cytotechnology.

[17] Kim T., Kim H.J., Cho S.K., Kang W.Y., Baek H., Jeon H.Y., Kim B., Kim D. (2011). *Nelumbo nucifera* extracts as whitening and anti-wrinkle cosmetic agent. Korean J. Chem. Eng..

[18] Moon S.H., Kim E., Kim H.-I., Kim S.-Y., Seo H.-H., Lee J.H., Lee M.-S., Lee S.-K., Moh S.H., Bae S. (2023). Skin-whitening effect of a callus extract of *Nelumbo nucifera* isolate Haman. Plants.

[19] Zheng W., Chen R., Xu K., Wang R., Wang Z., Li H., Go Y., Chan X., Huang Q., Wu J. (2025). Flavonoids in lotus stamen extract inhibit high glucose-induced intracellular glycation in fibroblasts by upregulating the expression of glyoxalase 1 and alleviating oxidative stress. Antioxidants.

[20] Lin H.-Y., Kuo Y.-H., Lin Y.-L., Chiang W. (2009). Antioxidative effect and active components from leaves of lotus (*Nelumbo nucifera*). J. Agric. Food Chem..

[21] Niranjan P.S., Maurya V.B., Swarnkar R.J., Singh D. (2023). Antimicrobial activity of *Nelumbo nucifera* leaves. Int. J. Biol. Pharm. Allied Sci..

[22] Shen Q., Wang J., Yao N., Niu X., Liu M., Li X. (2025). Hepatoprotective effect of lotus leaf against non-alcoholic fatty liver disease in rats via alteration of AMPK/SIRT1 and Nrf2/HO-1 signaling pathway. Acta Cir. Bras..

[23] Su Z.-Y., Lai B.-A., Lin Z.-H., Wei G.-J., Huang S.-H., Tung Y.-C., Wu T.-Y., Hun Lee J., Hsu Y.-C. (2022). Water extract of lotus leaves has hepatoprotective activity by enhancing Nrf2- and epigenetics-mediated cellular antioxidant capacity in mouse hepatocytes. J. Funct. Foods.

[24] Du H., You J.-S., Zhao X., Park J.-Y., Kim S.-H., Chang K.-J. (2010). Antiobesity and hypolipidemic effects of lotus leaf hot water extract with taurine supplementation in rats fed a high fat diet. J. Biomed. Sci..

[25] Wu Y., Tan F., Zhang T., Xie B., Ran L., Zhao X. (2020). The anti-obesity effect of lotus leaves on high-fat-diet-induced obesity by modulating lipid metabolism in C57BL/6J mice. Appl. Biol. Chem..

[26] Thu H.T.H., Xuan P.T.T., Hang N.T.T., Vu N.N., Phuoc T.H. (2016). Hypoglycemic effect of lotus (*Nelumbo nucifera* Gaertn.) flower ethanolic extract on alloxan induced diabetes rat model. Ho Chi Minh City Open Univ. J. Sci..

[27] Xu H., Wang C., Gong L. (2024). Hypoglycemic activity in vivo and in vitro of the lotus (*Nelumbo nucifera* Gaertn.) seed skin (Testa) phenolic-rich extracts. Food Chem. X.

[28] He S., Wang C., Shi W., Zhou C., Liu C., Xu Y., Gui Y., Jiang X., Fan G. (2025). Assessment of the properties and mechanism of three lotus (*Nelumbo nucifera* Gaertn.) parts for sleep improvement. Chem. Biodivers..

[29] Zhu M.-Z., Wu W., Jiao L.-L., Yang P.-F., Guo M.-Q. (2015). Analysis of flavonoids in lotus (*Nelumbo nucifera*) leaves and their antioxidant activity using macroporous resin chromatography coupled with LC-MS/MS and antioxidant biochemical assays. Molecules.

[30] Lee J.S., Paje L.A., Choi W.-H., Cho E.J., Kim H.Y., Jacinto S.D., Lee S. (2020). Validation of an optimized HPLC/UV method for the quantification of flavonoids in lotus. Appl. Biol. Chem..

[31] Chen S., Fang L., Xi H., Guan L., Fang J., Liu Y., Wu B., Li S. (2012). Simultaneous qualitative assessment and quantitative analysis of flavonoids in various tissues of lotus (*Nelumbo nucifera*) using high performance liquid chromatography coupled with triple quad mass spectrometry. Anal. Chim. Acta.

[32] Sahu B., Sahu M., Sahu M., Yadav M., Sahu R., Sahu C. (2024). An updated review on *Nelumbo nucifera* Gaertn: Chemical composition, nutritional value and pharmacological activities. Chem. Biodivers..

[33] Park C.H., Yeo H.J., Baskar T.B., Park Y.E., Park J.S., Lee S.Y., Park S.U. (2019). In vitro antioxidant and antimicrobial properties of flower, leaf, and stem extracts of Korean mint. Antioxidants.

[34] Abeysinghe D.C., Li X., Sun C., Zhang W., Zhou C., Chen K. (2007). Bioactive compounds and antioxidant capacities in different edible tissues of citrus fruit of four species. Food Chem..

[35] Jing L., Ma H., Fan P., Gao R., Jia Z. (2015). Antioxidant potential, total phenolic and total flavonoid contents of *Rhododendron anthopogonoides* and its protective effect on hypoxia-induced injury in PC12 cells. BMC Complement. Altern. Med..

[36] Dhanaraj F.I., Kalimuthu J.K., Balamurugan P.S., Subramani P., Katerere D.R., Gurusamy M. (2025). Investigating the phytochemical profile and antioxidant activity of different solvent extracts of *Sesamum prostratum* Retz. Plants.

[37] Thaipong K., Boonprakob U., Crosby K., Cisneros-Zevallos L., Hawkins Byrne D. (2006). Comparison of ABTS, DPPH, FRAP, and ORAC assays for estimating antioxidant activity from guava fruit extracts. J. Food Compos. Anal..

[38] Rana A., Samtiya M., Dhewa T., Mishra V., Aluko R.E. (2022). Health benefits of polyphenols: A concise review. J. Food Biochem..

[39] Schinella G.R., Tournier H.A., Prieto J.M., Mordujovich de Buschiazzo P., Ríos J.L. (2002). Antioxidant activity of anti-inflammatory plant extracts. Life Sci..

[40] Parham S., Kharazi A.Z., Bakhsheshi-Rad H.R., Nur H., Ismail A.F., Sharif S., RamaKrishna S., Berto F. (2020). Antioxidant, antimicrobial and antiviral properties of herbal materials. Antioxidants.

[41] Rodríguez-Yoldi M.J. (2021). Anti-inflammatory and antioxidant properties of plant extracts. Antioxidants.

[42] Jang J.Y., Ahn J.H., Jo Y.H., Hwang B.Y., Lee M.K. (2019). Antioxidant activity and phenolic content of different parts of lotus and optimization of extraction condition using response surface methodology. Nat. Prod. Sci..

[43] Palaiogiannis D., Chatzimitakos T., Athanasiadis V., Bozinou E., Makris D.P., Lalas S.I. (2023). Successive solvent extraction of polyphenols and flavonoids from *Cistus creticus* L. Leaves. Oxygen.

[44] Dias M.C., Pinto D.G.C.A., Silva A.M.S. (2021). Plant flavonoids: Chemical characteristics and biological activity. Molecules.

[45] Cheng Y., Xue F., Yang Y. (2023). Hot water extraction of antioxidants from tea leaves—Optimization of brewing conditions for preparing antioxidant-rich tea drinks. Molecules.

[46] Nutho B., Tungmunnithum D. (2023). Exploring major flavonoid phytochemicals from *Nelumbo nucifera* Gaertn. as potential skin anti-aging agents: In silico and in vitro evaluations. Int. J. Mol. Sci..

[47] Jung S.K., Lee K.W., Kim H.Y., Oh M.H., Byun S., Lim S.H., Heo Y.-S., Kang N.J., Bode A.M., Dong Z. (2010). Myricetin suppresses UVB-induced wrinkle formation and MMP-9 expression by inhibiting Raf. Biochem. Pharmacol..

[48] Huang J.-H., Huang C.-C., Fang J.-Y., Yang C., Chan C.-M., Wu N.-L., Kang S.-W., Hung C.-F. (2010). Protective effects of myricetin against ultraviolet-B-induced damage in human keratinocytes. Toxicol. In Vitro.

[49] Lee D.H., Lee C.S. (2016). Flavonoid myricetin inhibits TNF-α-stimulated production of inflammatory mediators by suppressing the Akt, mTOR and NF-κB pathways in human keratinocytes. Eur. J. Pharmacol..

[50] Xie J., Zheng Y. (2017). Myricetin protects keratinocyte damage induced by UV through IκB/NFκb signaling pathway. J. Cosmet. Dermatol..

[51] Gao J.-F., Tang L., Luo F., Chen L., Zhang Y.-Y., Ding H. (2023). Myricetin treatment has ameliorative effects in DNFB-induced atopic dermatitis mice under high-fat conditions. Toxicol. Sci..

[52] Zhang X., Li J., Liu Y., Ma R., Li C., Xu Z., Wang R., Zhang L., Zhang Y. (2025). Myricetin alleviates DNCB-induced atopic dermatitis by modulating macrophage M1/M2 polarization. Int. Immunopharmacol..

[53] Ha A.T., Rahmawati L., You L., Hossain M.A., Kim J.-H., Cho J.Y. (2021). Anti-inflammatory, antioxidant, moisturizing, and antimelanogenesis effects of quercetin 3-O-β-D-glucuronide in human keratinocytes and melanoma cells via activation of NF-κB and AP-1 pathways. Int. J. Mol. Sci..

[54] Raghunath A., Sundarraj K., Nagarajan R., Arfuso F., Bian J., Kumar A.P., Sethi G., Perumal E. (2018). Antioxidant response elements: Discovery, classes, regulation and potential applications. Redox Biol..

[55] Surh Y.J., Chun K.S., Cha H.H., Han S.S., Keum Y.S., Park K.K., Lee S.S. (2001). Molecular mechanisms underlying chemopreventive activities of anti-inflammatory phytochemicals: Down-regulation of COX-2 and iNOS through suppression of NF-κB activation. Mutat. Res..

[56] Zhong R., Miao L., Zhang H., Tan L., Zhao Y., Tu Y., Angel Prieto M., Simal-Gandara J., Chen L., He C. (2022). Anti-inflammatory activity of flavonols via inhibiting MAPK and NF-κB signaling pathways in RAW264.7 macrophages. Curr. Res. Food Sci..

